# Epigenetic Regulation of Adipogenic Differentiation by Histone Lysine Demethylation

**DOI:** 10.3390/ijms20163918

**Published:** 2019-08-12

**Authors:** Geovanny I. Nic-Can, Beatriz A. Rodas-Junco, Leydi M. Carrillo-Cocom, Alejandro Zepeda-Pedreguera, Ricardo Peñaloza-Cuevas, Fernando J. Aguilar-Ayala, Rafael A. Rojas-Herrera

**Affiliations:** 1 CONACYT-Facultad de Ingeniería Química, Universidad Autónoma de Yucatán.; Periférico Norte Kilómetro 33.5, Tablaje Catastral 13615, Chuburná de Hidalgo Inn, Mérida 97203, Yucatán, Mexico; 2 Laboratorio Translacional de Células Troncales-Facultad de Odontología, Universidad Autónoma de Yucatán, Calle 61-A X Av, Itzaes Costado Sur “Parque de la Paz”, Col. Centro, Mérida 97000, Yucatán, Mexico; 3 Facultad de Ingeniería Química, Universidad Autónoma de Yucatán.; Periférico Norte Kilómetro 33.5, Tablaje Catastral 13615, Chuburná de Hidalgo Inn, Mérida 97203, Yucatán, Mexico

**Keywords:** adipogenesis, cell differentiation, epigenetics, histone lysine demethylases, mesenchymal stem cells

## Abstract

Obesity is a rising public health problem that contributes to the development of several metabolic diseases and cancer. Adipocyte precursors outside of adipose depots that expand due to overweight and obesity may have a negative impact on human health. Determining how progenitor cells acquire a preadipocyte commitment and become mature adipocytes remains a significant challenge. Over the past several years, we have learned that the establishment of cellular identity is widely influenced by changes in histone marks, which in turn modulate chromatin structure. In this regard, histone lysine demethylases (KDMs) are now emerging as key players that shape chromatin through their ability to demethylate almost all major histone methylation sites. Recent research has shown that KDMs orchestrate the chromatin landscape, which mediates the activation of adipocyte-specific genes. In addition, KDMs have functions in addition to their enzymatic activity, which are beginning to be revealed, and their dysregulation seems to be related to the development of metabolic disorders. In this review, we highlight the biological functions of KDMs that contribute to the establishment of a permissive or repressive chromatin environment during the mesenchymal stem cell transition into adipocytes. Understanding how KDMs regulate adipogenesis might prompt the development of new strategies for fighting obesity-related diseases.

## 1. Introduction

Obesity has become a serious and increasing public health problem. According to the World Health Organization, its prevalence has tripled over the past 40 years. In 2016, an estimated 39% of adults aged 18 years were overweight, and 13% were obese [1]. This worldwide epidemic is the result of a combination of several factors, such as genetic susceptibility (a nucleotide polymorphism within the fat mass and obesity-associated gene (FTO)), sedentary lifestyles, and overconsumption of high-energy foods that exceed the caloric necessity of the individual [2,3]. These factors promote excessive lipid accumulation in white adipose tissue (WAT) and results in an increase in the volume and number of adipocytes [4]. Pathologically, obesity increases the risk of developing insulin resistance, type 2 diabetes mellitus, skin disorders, cardiovascular diseases, asthma, and some types of cancer [5,6]. In these cases of disease, the metabolic pathologies associated with overweight and obesity, combined with the emotional and psychosocial problems they invoke, increase health-care costs. In the United States alone, these costs could reach between 48 and 66 billion dollars per year by 2030 [1,2,5]. Therefore, due to the growing epidemic and the rising costs of preventing the health consequences associated with obesity, a deep understanding of the molecular mechanisms underlying adipocyte biology, including the transition from stem cells to adipocyte development, is expected to play a critical role in combating obesity.

**Figure 1:**
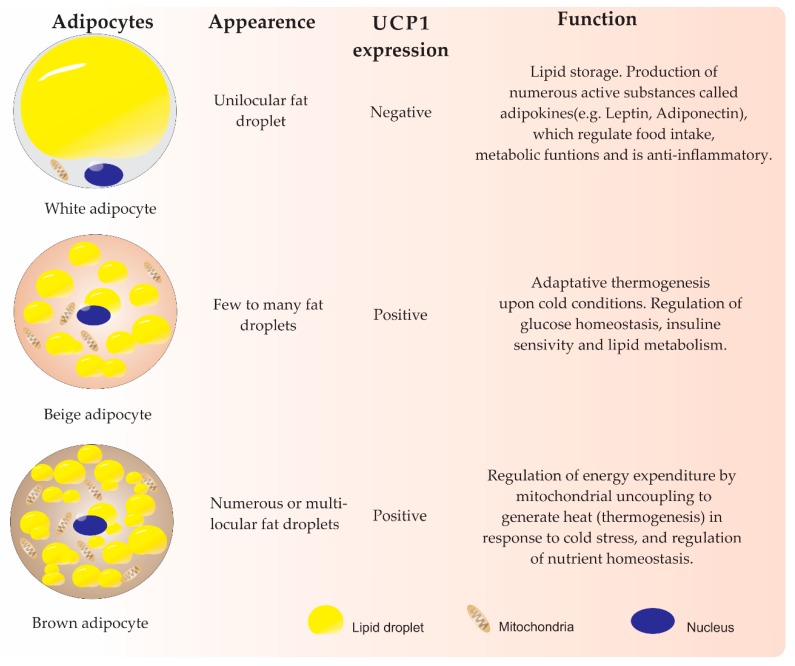
Properties of adipose tissue [2,4,5,10,11,12,13,15,22,23].

In mammals, adipose tissue plays an essential role in maintaining energy and metabolic homeostasis. This tissue has been classified into two main types by color: WAT and brown adipose tissue (BAT). WAT contains the classic fat cells responsible for storing lipids within one large unilocular droplet and releases them (Figure 1). White adipose cell populations are distributed throughout the body, forming adipose depots mainly in visceral and subcutaneous regions that can expand with obesity [2,7]. It has been shown that visceral adipose tissue (VAT) contains a higher percentage of inflammatory cells and immune cells, and it has more cellular, more vasculature, and greater innervation than subcutaneous WAT (scWAT); as a result of these features, VAT has been associated with negative metabolic effects [8,9]. In another feature that differs from VAT, scWAT has great adipogenic potential. Furthermore, upon cold exposure or β-adrenergic stimulation, some adipocytes from scWAT may acquire features of brown adipocytes in a process referred to as “browning” or “beiging” of white fat [10,11]. These cells, known as beige or brite adipocytes, exhibit a medium level of mitochondria density, have few or multilocular lipids, and acquire the ability to express uncoupling protein 1 (*UCP1*) [11,12,13]. Therefore, beige adipocytes have drawn much attention due to their beneficial functions in combating obesity by increasing thermogenesis, lipid oxidation, insulin sensitivity, and glucose tolerance [9,14]. However, whether the cells appear due to the transdifferentiation of white adipocytes and/or from progenitor cells residing in WAT remains uncertain [10,15,16].

On the other hand, BAT contains adipocytes composed of numerous lipid droplets and has high mitochondria density. BAT represents a discrete depot of tissue localized in the supraclavicular and periadrenal regions that specialize in metabolizing fatty acids to produce heat (thermogenesis) through the BAT-selective expression of *UCP1* [10,17,18]. In obese individuals, BAT activity decreases, mainly because of the conversion of brown adipocytes to unilocular white cells in a poorly known process called whitening. This conversion is stimulated by multiple factors, including high ambient temperature, leptin receptor deficiency, β-adrenergic receptor signal impairment, and lipase deficiency. WAT expands and can become dysfunctional, leading to a low-grade inflammatory state [19].

In particular, metabolic complications during obesity have been associated with limitations of subcutaneous white adipocytes to recruit and stimulate the differentiation of new adipocytes from precursor cells. It is known that if the adipose tissue cannot expand through adipogenesis, then the accumulated lipids produce adipocyte hypertrophy, causing the cells to rupture and enabling the invasion of macrophages along with the release of proinflammatory adipokines and the inhibited release of anti-inflammatory adipokines. This process leads to a proinflammatory and insulin-resistant state. In addition, adipocytes may be preferentially deposited in other organs, such as the liver, heart, kidney and pancreas, in a process called “ectopic fat deposition” [20,21]. Thus, adipogenesis is an important process in the regulation of obesity.

Despite morphological and functional differences, both white and brown adipocytes share similar features of differentiation. For instance, under appropriate stimuli, mesenchymal stem cells (MSCs) give rise to adipocytes through adipogenesis, which is a sophisticated and highly orchestrated program of gene expression that is widely influenced not only by genetic and environmental cues but also by epigenetic events [16,24]. In brief, the early adipogenic program, triggered by adipogenic stimuli, activates the expression of CCAT/enhancer-binding protein (EBP) family members (C/EBP-β and C/EBP-δ) and Krüppel-like factors (KLF4 and KLF5), which in turn activate the expression of peroxisome proliferator-activated receptor-γ (PPARγ) and C/EBP-α, leading to the induction of genes related to the adipogenic phenotype [25]. In contrast, canonical WNT/β-catenin signaling has been shown to inhibit adipogenesis while promoting osteogenesis [26,27,28].

**Figure 2:**
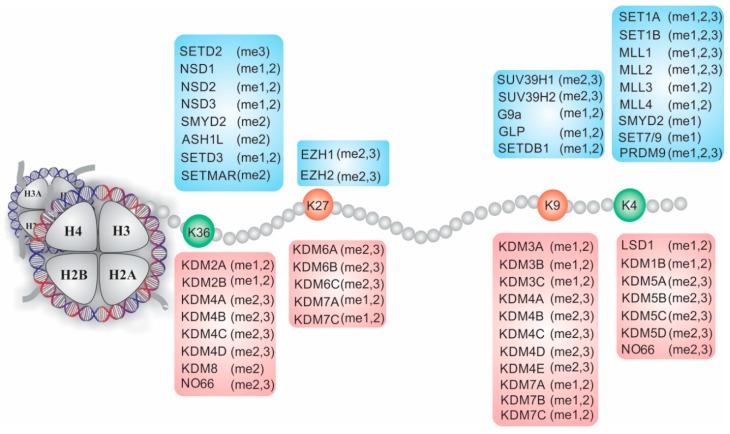
Nucleosome structure and dynamic modulation of lysine methylation on histone H3 mediated by histone lysine methyltransferases and demethylases (KDMs).

Regulation of the MSCs transition into a specific lineage cell is associated with multiple levels of epigenetic layers that contribute to the establishment of exclusive genetic programs. This process is due to several prevalent epigenetic modifications, including covalent modifications, such as methylation of nucleic acids and acetylation, methylation, phosphorylation and/or ubiquitination of histones, and the effects of small noncoding RNAs [29]. These modifications, also referred to as epigenetic marks, do not change the DNA sequence but can define the architecture and accessibility of chromatin and are reversible in response to several stimuli. Thus, combinations of these epigenetic modifications modulate chromatin structure and facilitate the expression of genes specific to a cell type, whereas they repress the transcription of fat genes of alternative lineages [30]. In this regard, histone lysine methylation is one of the best processes to demonstrate the influences of epigenetic function. Three forms of methylation on different lysine residues along the amino acid sequence of histone H3, each with a specific function, recruit reading and erasing proteins that activate or repress transcription at the appropriate time (Figure 2). Considering that epigenetic processes vary widely according to the cell, stage of cell development and specific tissue, their roles in the onset of adipogenesis remain unknown such that understanding the processes presents a significant challenge. In this review, we provide an overview of adipocyte development, giving particular attention to lysine histone demethylases and their role in the chromatin reconfiguration that induces MSCs to develop into adipocytes.

## 2. Adipogenesis Differentiation from MSCs

Currently, tissue-specific stem cells can be isolated from bone marrow, dental pulp, liver and adipose tissue, and the placenta, among other sources [31,32]. These stem cell populations can undergo commitment and thus can mature into one of several cell lineages, including chondrocytes, myocytes, osteoblasts, and adipocytes. Additionally, MSCs also possess immunomodulatory function and low immunogenicity, making them a promising source for therapeutic or regenerative treatments. However, expectations from a broad base of clinical applications have not been realized. For instance, restoration of bone marrow MSCs (BMSCs) to stimulate their commitment into osteoblasts instead of adipocytes, thereby avoiding bone mass loss during osteoporosis, has not yet been possible [33,34]. In addition, no current therapeutic strategies have been able to reduce or prevent ectopic fat deposition in skeletal and/or cardiac muscle, liver or kidney, and the consequences of untreated deposition could potentially increase with obesity and negatively affect tissue function [2,5]. Therefore, a deeper understanding of the molecular mechanism underlying adipocyte biology is expected to reveal a strategy for combating obesity and related diseases.

To date, most of our knowledge about the molecular mechanisms that define adipocyte biology is derived from studies in vitro that used different preadipocyte mouse cell line models (3T3-L1 and 3T3-F442A cell lines) [35] and the occasional human primary cell culture, including those generated from bone marrow, umbilical cord, and adipose tissue [32]; human embryonic stem cells (hESCs); or induced pluripotent stem cells (iPSs) [36]. In these cases, the elucidation of the molecular mechanisms involved in adipogenic differentiation is limited because the process in vivo occurs in tissues formed by a mixture of cells, such as macrophages, fibroblasts, and endothelial cells, among others. Therefore, it is still uncertain whether these models in vitro represent the real characteristics of adipogenic precursors [21,37]. At present, a growing body of evidence indicates that multiple signals are directly involved in the onset of adipogenesis and that this process involves two phases: Determination and terminal differentiation. The first phase involves the conversion of MSCs into preadipocytes, and although they are indistinguishable morphologically from the precursor cells, they have lost the ability to differentiate into another cell type. In the second phase, the preadipocyte cell becomes ovoid/round in morphology and acquires the traits of the mature adipocyte, including the ability to synthesize and transport lipids, secrete adipocyte-specific proteins, and express the machinery for insulin sensitivity [4,38,39]. In addition, extensive studies have revealed that adipogenesis is a highly complicated process that depends on the integration of multiple signals, including bone morphogenetic proteins (BMP2 and BMP4), hedgehog and WNT/*β*-catenin signaling, insulin growth factor (IGF), transforming growth factor-*β* (TGFβ), and fibroblast growth factors (FGF1 and FGF2), among others (reviewed elsewhere [35,40]). All these cues have both activating and inhibitory roles and often work mutually to modulate the cell fate of MSCs commitment to adipocyte progenitor cells.

On the other hand, a series of excellent reviews have provided details on the transcriptional program required for adipocyte differentiation [35,41,42]. Briefly, under adipogenic induction conditions, inducers such as insulin and dexamethasone activate the insulin growth factor 1 (IGF1), glucocorticoid, and cyclic AMP (cAMP)-signaling pathways. An elevated level of cAMP induces the phosphorylation of cAMP response element-binding protein (CREB) that, in turn, enables the expression of *C/EBPβ*, which encodes a protein product requiring dual phosphorylation by a MAP kinase and GSK3β to produce a conformational change that activates its DNA-binding activity. Once active, C/EBPβ can bind in proximity to the promoters of *PPARγ* and *C/EBPα* and upregulate their expression [43]. PPARγ and C/EBPα, the most important players in adipogenesis, work together to activate multiple downstream adipocyte-specific genes (including those that induce triglyceride synthesis and lipid storage) for acquisition of the mature adipocyte phenotype [25,44]. Moreover, several KLF proteins (KLF4-6, 9 and 15) [17] and sex determining region Y-box2 (SOX2) [45], which are transcription factors (TFs) involved in the maintenance of stemness, self-renewal, and the control of cell proliferation and differentiation, are variably expressed during adipogenesis. More recently, genome-wide transcriptome analysis has revealed an increasing number of TFs that are differentially expressed during commitment from human MSCs to cells of adipogenic lineage [46,47]. For instance, the results from an expression analysis highlighted three phases during which BMSCs adopt a committed adipocyte phenotype: initiation of differentiation, lineage acquisition, and early lineage progression. It is worth noting that, at the beginning of differentiation, there is a burst of expression changes in many transcription-related genes that regulate lineage commitment and prime cells to acquire a specialized mesenchymal phenotype [47]. However, to acquire a better understanding of adipocyte biology, further studies are needed to reveal how normally repressed genes are activated during cell lineage commitment. In addition, it will be interesting to use three-dimensional cultures and cocultures as models to extend the knowledge of adipogenic differentiation by studying their relationships with the cells surrounding them in vivo and within an environment that mimics the complexity of organs [37].

## 3. Epigenetic Regulation of Gene Expression

Despite substantial progress made in determining the molecular basis of adipogenesis, we still have limited information about the chromatin-based processes that regulate the activation or repression of TF-encoding genes during adipogenic commitment. However, it is known that cell differentiation can be modulated by epigenetic processes that determine how and when lineage-specific genes are expressed. Unlike fixed DNA, reversible covalent chemical modifications on DNA and histones can change chromatin conformation at specific developmental stages to establish the unique chromatin landscapes associated with adipogenesis [16]. To date, a dozen histone posttranslational modifications (PTMs) and several DNA modifications have been identified, with DNA methylation and the acetylation and methylation of histones being the most studied [29]. However, methylation is considered the most complicated epigenetic mark since it affects both DNA and histones [48]. In the case of DNA, for instance, methylation is catalyzed by DNA methyltransferases, which transfer a methyl group at position C5 of 2′-deoxycytosine in the dinucleotide CpG, a process that can be reversed through demethylation mediated by ten eleven translocation enzymes [49]. Thus, DNA-modifying enzyme methylation plays an essential role in gene regulation and cellular function.

Although the canonical histones (H2A, H2B, H3 and H4) have PTMs, some modifications simply induce or repress expression (e.g., H3K9 acetylation or H2A ubiquitination, respectively) [50,51]. In contrast, histone H3 lysine methylation (H3Kme) is a complex modification since it can occur in the monomethyl (me1), dimethyl (me2) or trimethyl (me3) state. In this regard, different methylation states confer functional diversity depending on their position. For example, H3K4me3, H3K36me3 and H3K79me3 mark active transcription, whereas H3K9me2/3 and H3K27me3 are marks related to repressed genes [50,52]. The type of methylation that occurs is determined by several histone lysine methyltransferases (KMTs); however, methylation can be reversed by diverse histone lysine demethylases (KDMs) (Figure 2), and thus, the methylation states of chromatin elements are balanced [51,53]. In addition to findings on regulating transcriptional events, several genome-wide studies have demonstrated that histone lysine methylation is dynamically regulated during embryogenic development, cell cycle processes, cell fate decisions, and DNA replication and repair and is observed in a wide range of human diseases, suggesting that KMTs and KDMs play critical roles during these processes [53,54]. Here, we focus on recent studies based on methylation by KMTs and how these methylation states are reshaped by KDMs to help establish a permissive or inhibitory chromatin environment during the mesenchymal stem cell transition into adipocytes. However, this topic will be discussed only in relation to the biogenesis of white adipocytes or the browning of white fat since the relationship between KDMs and beige adipocyte differentiation has not been studied extensively [55].

## 4. The Role of KMTs in the Adipogenic Differentiation of MSCs

Adipocyte differentiation occurs rapidly after adipogenic induction, which is tightly controlled by the diverse transcriptional activity required to convert human BMSCs into mature adipocytes. For instance, more than 3800 genes are differentially upregulated 3 days after adipogenic induction, suggesting that transient lineage-specific stimulation and suppression of early responder TFs may represent a precommitment stage that drives the activation of the target genes to establish the phenotypes necessary to support adipocyte metabolism [47]. Genome-wide mapping studies have shown that adipogenesis proceeds through dynamic chromatin reconfiguration [56,57]. By using a DNase I hypersensitivity assay, it was shown that C/EBP-β binds to regulatory element hotspots before induction and chromatin remodeling to sustain adipogenesis onset and development [57]. More than 16,000 C/EBPβ binding sites have been found in the closed chromatin of preadipocytes, and they facilitate recruitment of glucocorticoid receptors (GR), retinoid X receptors (RXR) and signal transducers and activators of transcription 5A (STAT5A) to generate an active epigenomic landscape as well as to activate nearby genes during early adipogenesis [58]. Xiao et al. [59] reported that the *PPARγ* promoter is remodeled within 2 h after adipogenic induction in 3T3-L1 cells through a process that depends on C/EBPβ; however, this gene is expressed only in the later stages of adipogenesis. These results suggest that changes in chromatin accessibility take place before the transcriptional activation of *PPARγ*. Studies using chromatin immunoprecipitation coupled to DNA microarrays (ChIP-chip) and ChIP-sequencing identified extensive colocalization of C/EBPα and PPARγ at several enhancer regions where they cooperate directly with chromatin to activate thousands of genes related to adipogenic phenotypes [44]. To set up the adipogenic environment, these enhancer regions are enriched with histone marks related to open chromatin and transcriptional activation, including acetylated H3K27 (H3K27ac), H3K4me1/2/3, and H3K36me2 [56,60,61]. It has been observed that both the H3K4 monomethyltransferase and dimethyltransferase mixed-lineage leukemia 3-4 (MLL3 and MLL4, respectively) directly promote *PPARγ* expression. During the early phase of adipogenesis, C/EBPβ recruits MLL3/MLL4 to increase the levels of H3K4me1/2 and establish adipogenic enhancers at the *PPARγ* and *C/EBPα* gene loci. In contrast, deletion of both MLL3 and MLL4 markedly decreases the effects of H3K4me1/2 and H3K27ac on enhancers, thereby impairing adipogenesis development [62]. These findings have been recently confirmed, indicating that MLL3 and MLL4 are necessary to facilitate CBP/300 binding to activate enhancers through H3K27ac. These proteins are recognized by Brd4, an epigenomic reader that recruits a transcription coactivator Mediator and Pol II to activate cell-identity-specific gene expression during adipogenesis [63]. Moreover, it has also been reported that many PPARγ-binding sites at enhancer regions are enriched with high levels of H3K4me3 [60], which likely serve to properly define the adipogenic program driven by PPARγ and C/EBP.

More recently, two important studies showed that a genetic mutation in which lysines 4 and 36 are substituted by methionine on histone H3.3 (H3.3K4M and H3.3K36M, respectively) inhibits adipogenesis [61,64]. Jang et al. showed that the ectopic expression of H3.3K4M destabilizes MLL3/MLL4 proteins such that activation by enhancers is suppressed during adipogenesis [64]. Although H3.3K4M does not affect adipose tissue maintenance, loss of the enzymatic SET domain of MLL3/MLL4 in mice revealed that this domain is essential for proper fatty tissue development. In addition, H3.3K4M expression decreased global levels of H3K4me1/2/3, suggesting that this mutation inhibits the enzymatic functions of multiple KMTs, including MLL3 and MLL4; however, the biochemical mechanisms for this mediation are still unknown. Similar to that of H3.3K4M, the loss of H3K36 methylation of H3.3K36M also impaired adipogenic induction [61]. Mechanistically, the ectopic expression of H3.3K36M depleted the levels of H3K36me2 and increased those of H3K27me3 at the *PPARγ* and *C/EBPα* gene loci, inhibiting the expression of these genes. As a consequence, the development of both white and brown adipocytes was profoundly inhibited. By using knockdown screening, the same research group determined that, during loss of Nsd2 function, the KMT that generates H3K36me1/2, but not the major H3K36 trimethyltransferase Setd2, mimics the phenotype of H3.3K36M. Although there are two KMTs for H3K36me2, Nsd1 and Nsd2 [65], the *Nsd2* knockdown had prominent effects on the decrease in H3K36me2 levels at the *C/EBPα*, *Lpl*, and *CD36* (also known as fatty acid translocase) loci, suggesting that Nsd2 has a specific role in regulating adipogenesis-related genes [61].

In preadipocytes, it has been shown that different KMTs generate chromatin marks that promote the transcriptional repression of *PPARγ* but that these marks are lost during adipocyte differentiation [66,67]. For instance, Wang et al. revealed that G9a, the main histone methyltransferase responsible for generating H3K9me2, is selectively enriched at the entire *PPARγ* gene locus [67]. Consistent with this finding, G9a and H3K9me2 levels are decreased at the *PPARγ* locus during adipocyte differentiation. In addition, deletion of G9a promotes chromatin remodeling by increasing H3K9ac, which facilitates chromatin opening and recruitment of C/EBPβ to the proximal *PPARγ2* promoter to enabling its expression. Interestingly, the same authors also showed that G9a promotes *WNT10a* gene expression independent of its enzymatic activity. These results indicate that G9a suppresses adipogenesis by inhibiting *PPARγ* and inducing *WNT10a* (an osteogenesis-related gene) expression [67]. Furthermore, SET domain bifurcated 1 (SETDB1), another H3K9me2/3 methyltransferase, also appears to act as an inhibitor of adipogenesis [66,68]. In this case, after phosphorylation by nemo-like kinase, SETDB1 is recruited as part of a corepressor complex that catalyzes H3K9me2 of the *PPARγ* target gene promoter to inactivate *PPARγ* expression [66]. Consistent with its function, knocking down *SETDB1* decreases H3K9me2 levels at the *C/EBPα* promoter and promotes adipogenesis development [68].

In ESCs, the combination of active and repressive histone marks (H3K4me3 and H3K27me3) at the same promoter, referred to bivalent domains, poises developmental genes for expression for timely activation while maintaining repression in the absence of signals to differentiate [69]. Current evidence suggests that lineage-specific gene-body DNA methylation recruits SETDB1 to form a noncanonical H3K4me3/H3K9me3 chromatin bivalent domain in cells committed to the adipocyte lineage, including 10T1/2 MSCs and 3T3-L1 preadipocytes [70]. Mechanistically, the methyl binding domain (MBD) and MBD-containing chromatin-associated factor-1 (MCAF1) are required for SETDB1 recruitment to m5CpG sites at the *C/EBPα* and *PPARγ* loci, where trimethylation of H3K9 is immediately catalyzed at the transcription start sites, which is marked with H3K4me3 to modulate gene expression. This mechanism generates a single euchromatin/heterochromatin border that restricts C/EBPβ binding and suppresses H3K27ac action, pausing RNA pol II activity and limiting *C/EBPα* and *PPARγ* expression and adipogenesis [70]. However, under proper differentiation stimuli or *SETDB1* knockdown, this border can be removed to favor euchromatin by reducing H3K9me3 levels, thereby enabling C/EBPβ binding, H3K27ac, RNA pol II elongation and gene expression [70]. This finding suggests that bivalent chromatin domain plays a crucial role in fine-tuning adipogenesis development.

In contrast to the chromatin bivalent domain, an inverse correlation between H3K9me2 and H3K27me3 has also been identified in preadipocytes. For instance, whereas H3K9me2 levels are higher at the *PPARγ* promoter, in genes related to osteogenesis, such as *Wnt1*, *Wnt6*, and *Wnt10a*, H3K9me2 levels are very low, and the opposite is also true: H3K27me3 is enriched at the loci of *Wnt* genes but absent at the *PPARγ* locus [26,67]. Ge et al. revealed that enhancer of zeste homolog 2 (EzH2) is required to facilitate adipogenesis [26]. EzH2, a member of the polycomb repressive complex 2 (PRC2), catalyzes the deposition of H3K27me3, which is recognized by PRC1 to promote gene silencing [71]. EzH2 binds to the proximal promoter of *Wnt* genes, increasing the levels of H3K27me3 to directly repress *Wnt1*, *Wnt6*, *Wnt10a* and *Wnt10b* in preadipocytes and during adipogenesis. In addition, *GATA* and *Pref-1*, two inhibitors of adipogenesis, are also direct targets of EzH2 [26]. In contrast, the loss of function of EzH2 leads to the activation of Wnt/β catenin signaling by reducing H3K27me3 at the promoters of *Wnt* genes, which in turn diminishes the binding of the proto-oncogene polycomb ring finger (Bmi1), the major component of PRC1 [72], thus preventing transcriptional repression [26]. Additionally, transcription was upregulated by increasing levels of H3K27ac at the proximal promoters of *Wnt* genes [26]. Together, these mechanisms resulted in the suppression of several adipogenic markers, including *PPARγ, C/EBP-α* and *FABP4,* resulting in severe white adipogenesis impairment. However, the phenotype was rescued by the ectopic expression of EzH2 of PPAR*γ* and inhibitors of Wnt/β catenin signaling [26]. Together, these results indicate that KMTs control the expression of positive and negative regulators implicated in the adipogenic transcriptional network. Thus, the establishment of proper lineage might depend on the integration of environmental stimuli and multiple levels of epigenetic control to guarantee the access of the adipogenic factors (e.g., PPARγ and C/EBPα) to DNA. Given that adipogenesis requires dramatic changes in chromatin remodeling, it would be interesting to know how the overexpression of *EzH2* might contribute to the establishment of enhancers that facilitate the adipogenic program. Under the same schema, it would also be interesting to know whether EzH2 overexpression increases H3K27me3 levels at the promoters of transcriptional factors from alternative lineages.

## 5. Histone Lysine Demethylases (KDMs)

As a vital element of the eukaryotic epigenome, the histone lysine methylation status can directly affect chromatin structure, thereby determining the transcription of the surrounding genes. Because of its low turnover rate, it has been thought for some time that lysine methylation is irreversible. However, the debate about its reversibility finally ended with the discovery of histone lysine demethylase 1 (KDM1 or LSD1) in 2004 [73]; it was the first protein found that removes methyl groups from H3K4me1/2, which it accomplishes through the amine oxidase domain using flavine adenine dinucleotide (FAD) as a cofactor. Shortly after this discovery, Zhang and colleagues highlighted the identification of KDM2A, also known as JHDM1A or FBXL11, which demethylates H3K36me1/2 via its JmjC domain through a 2-oxoglutarate-Fe^2+^-dependent dioxygenase reaction [74]. Currently, in humans, more than 30 different KDMs have been found that actively catalyze the demethylation of almost all histone methylation sites (H3K4, H3K9, H3K27, and H3K36). On the basis of emerging findings, it has been revealed that KDMs play an essential role in chromatin-based processes, including the regulation of the cell cycle, DNA repair, embryonic development, stem cell renewal, and differentiation [75,76], whereas altered demethylase activities have been associated with the development of several diseases and obesity (Table 1) [77,78,79]. Based on these observations, we discuss how KDMs create certain states in chromatins that could contribute to transcriptional activation or repression during the cell fate transition into adipocytes.

### The Role of KDMs during Adipogenesis

In all living organisms, epigenetic status defines the identity of every single cell. As a consequence, the epigenome needs to be reprogrammed in a concerted manner during the differentiation of each cell line. However, the epigenetic mechanisms underlying adipocyte biology have yet to be elucidated. The fact that adipogenic events can occur in different depots (e.g., liver, bone marrow, skeletal muscle, and the pericardium, among others) in addition to visceral and subcutaneous fat tissues implies that adipogenesis might be differentially regulated at different adipogenic sites [21]. This consideration adds complexity to study of the tissue origin, metabolic function, and functional properties of adipocytes. As adipocytes are found in multiple origins [15,21,80], more epigenetic studies are urgently needed to determine how histone modifications activate or repress the key TFs that induce cells toward adipocyte commitment. In this regard, KDMs can contribute to the establishment of a transcriptional chromatin state or impose gene repression to modulate cell fate differentiation [75]. For instance, the search for how and what kinds of KDMs regulate the adipogenic specification of MSCs led to the identification of KDM3A, a H3K9-specific demethylase, as an important epigenetic factor related to normal weight control in mice [78]. KDM3A is recruited to the PPAR responsive element (PPRE) of the *Ucp1* gene, not only to decrease H3K9me2 at the PPRE but also to facilitate the recruitment of PPARγ and RXRα and their coactivators to promote brown adipogenesis (BA). The lack of KDM3A activity leads to obesity in adult mice by disrupting BA and increasing fat droplets in WAT, as well as in muscle and liver. This process results in abnormal fat metabolism and obesity (Table 1) [78]. In a recent report, it was also shown that KDM3A senses environmental stress via signal-specific PTMs to revise the chromatin information for subcutaneous WAT, which leads to an adaptative phenotype (beige adipocytes) that can tolerate low temperatures. It appears that KDM3A must be phosphorylated at serine-265 through a β-adrenergic signal prior to the formation of the pS265-KDM3A-PPARγ-PGC1α-PRDM16 transcription complex that target beige-selective genes, including *Ucp1* and *Cidea*, among others, where H3K9me2 levels are reverted and lead to the beiging of subcutaneous WAT [81].

Another KDM that has been shown to regulate the adipogenic program is LSD1, which is a demethylase with dual targets: H3K4 and H3K9 [82]. In 3T3-L1 preadipocytes, LSD1 was shown to act by removing the repressive H3K9me2 mark deposited by SETDB1 at the *ADIPOQ* and *C/EBPα* promoters to favor the differentiation and function of adipocytes [68,83]. Given that the knockdown of *SETDB1* promotes adipogenic differentiation and that the loss of function of *LSD1* impairs adipocyte development, it is reasonable to suggest that LSD1 leads to a permissive chromatin state at both *ADIPOQ* and *C/EBPα* promoters by opposing the action of SETDB1 [68]. Moreover, it has also been observed that, whereas adipogenic genes decrease their expression, inflammatory genes [interleukin 6 (*IL6*), chemokine (C-C motif) ligand 2 (*CCL2*), chemokine (C-X-C motif) ligands 5 (*CXL5*) and *CXL10*] appear to be induced by decreased LSD1 activity, which leads to preadipocytes adopting a proinflammatory phenotype instead of a phenotype that is commensurate with mature adipocytes [22]; this phenotype is a feature of visceral WAT, where the obesity-induced release of proinflammatory adipokines leads to the development of several metabolic diseases, such as metabolic syndrome and diabetes mellitus [2]. In contrast to the effects of decreased *LSD1* levels, *LSD1* upregulation increases the mitochondrial biogenesis of WAT in mice that were exposed to cold by activating the expression of genes involved in oxidative phosphorylation [83]. Notably, while the catalytic activity of LSD1 is indispensable in the early stages of adipogenesis, it is not critical during late adipogenesis. In agreement with this observation, when preadipocytes of a stromal vascular fraction from transgenic mice (Tg) that overexpress *LSD1* and from control mice were undergoing adipogenesis, both expressed similar levels of adipogenic markers (*FAB4* and *ADIPOQ*); however, only the Tg adipocytes with elevated levels of LSD1 showed increased expression of thermogenic and oxidative markers. In addition, the increased levels of LSD1 in Tg mice promote the formation of islets in metabolically active brown-like adipocytes in WAT. Interestingly, these Tg specimens also showed limited weight gain even after ingesting a high-fat diet [83].

A recent finding also indicates that LDS1 cooperates with Zfp516 (a cold-induced transcriptional factor) to activate the BAT gene program. Mechanically, Zfp516 directly recruits LSD1 to the promoter regions of BAT genes (e.g., *Ucp1* and *Cidea*), which are required for the removal of H3K9me2 and the activation of thermogenesis and brown adipogenesis. In contrast, the loss of function of LSD1 leads to a significant gain in body weight in mice, since the lipid droplet size in brown adipocytes is increased, causing imbalanced BAT development and a decrease in energy expenditure [23].

On the other hand, LSD1 also regulates brown and beige adipocyte function not only by removing H3K9me2 but also by demethylating H3K4me2. To complete these functions, LSD1 interacts with PRDM16 (PR domain containing 16) to repress the expression of white fat selective genes in brown adipocytes, whereas adipose-specific LSD1 ablation impairs the fatty acid oxidation capacity of mitochondria and reduces energy expenditure, which leads to an increase in fat deposition [84]. These findings suggest that LSD1 specificity to orchestrate the demethylation of H3K9me2 at BAT loci, and H3K4me2 in WAT might be mediated via interactions with a variety of TFs. In addition to these findings, LSD1 is able to bind to the *Wnt* gene locus (e.g., *Wnt6a* and *Wnt10b*), where it demethylates H3K4me2 levels to promote BA differentiation. Whereas the inhibition or knockdown of *LDS1* impairs brown adipogenesis differentiation, the phenotype can be rescued by the Wnt/β catenin signaling inhibitor LGK974 [85]. Together, these new findings reveal that LDS1 is an additional epigenetic factor that, along with EzH2, suppresses components of the Wnt pathway to facilitate adipogenesis. Additionally, these findings on LSD1 modulation imply that LSD1 could be targeted as a treatment to stimulate energy expenditure or combat obesity-related diseases. In this regard, some studies claim that there is a link between LDS1 and beige adipogenesis; however, the role of the enzymatic activity of LSD1 during the beiging of white adipocytes remains to be elucidated [55,86].

However, whether LSD1 functions during differentiation in a stage-dependent manner remains unknown. Unlike mouse cells, in which deficient LSD1 impairs adipogenesis, in human ESCs, inhibition of LSD1 by CBB1007 leads to increasing *PPARγ2* and *C/EBPα* expression by increasing H3K4me2 levels, which enhances the adipogenic response [87]. This finding may mean that LDS1 suppressed the expression of adipogenesis-related genes through H3K4me2 demethylation since LSD1 maintains the silencing of several developmental genes in human ESCs [88]. Further studies are required to determine how LSD1 maintains ESC identity and how it supports the adipogenic differentiation program over time. It is also important to determine whether substrates are recognized in a development-dependent manner and whether this recognition mechanism is conserved among different human MSCs.

KDM7C, a jmjC known as plant homeodomain 2 (PHF2), also catalyzes the removal of a repressive H3K9me2 mark in a phosphorylation-dependent manner that is mediated by protein kinase A [89]. Okuno et al. showed that KDM7C interacts with C/EBP*α* to demethylate H3K9me2 at the promoters of *PPARγ* and *FABP4*, enabling their expression [90]. Consistent with these observations, the lack of KDM7C activity in preadipocytes inhibits overall adipogenesis gene expression and lipid formation, indicating that KDM7C coactivates TFs that promote adipogenesis [90].

On the other hand, KDM2A, an H3K36 demethylase [74], is required for proper cell cycle regulation and embryonic development, as shown in vivo in mice, in which inactivated KDM2A promoted cell death and embryonic lethality [91]. Unlike in mice, in humans, deficiency caused by *KDM2A* knockdown improves the adipogenic potential in the SCs from apical papilla (SCAPs) by increasing the expression of several adipogenic-related genes, including *C/EBPα*, *LPL, PPARγ*, and *CD36* [92]. In addition, depletion of *KDM2A* in SCAPs promoted H3K4me3 enrichment, but not that of H3K36me2, at the *NANOG* and *SOX2* promoters, improving the adipogenic potential. Consistently, higher SOX2 levels favor adipogenesis by inducing *PPARγ* and blocking osteogenesis [45]. However, whether KDM2A is an adipogenic repressor remains to be determined.

Growth arrest of preadipocytes and reentry into the cell cycle are among the main events required to generate the adipocyte phenotype. For instance, the number of 3T3-L1 cells increases up to 4-fold during adipogenic differentiation in a process known as mitotic clonal expansion (MCE) [93,94,95]. It has been shown that KDMs play an essential role in the control of cell cycle timing and dynamics by regulating the expression of several genes involved in cell cycle progression [75], as exemplified by the KDM2B, KDM4B, and KDM5 family members. In mice, the *KDM2B* gene can be found in three isoforms (*KDM2B1-3*) due to alternative promoters and splice sites. Among these isoforms, *KDM2B-*2 is an antiadipogenic factor predominantly induced during the early phase of adipogenic differentiation. Although KDM2B catalyzes the removal of H3K36me1/2 and H3K4me3 [76], this (the second) isoform lacks the JmjC demethylase domain. However, the overexpression of *KDM2B-2* leads to a substantial reduction in *C/EBPα*, and *PPARγ* expression. In addition, it arrested the entry of cells into S phase by suppressing *Cdk1-2* and Cyclin *E1* gene expression, suggesting that transcriptional inhibition is mediated by a mechanism other than demethylation at the catalytic JmjC domain [96]. Engineered mutants lacking the F-BOX and leucine-rich repeat (LRRs) domains of KDM2B enabled the identification of these domains as responsible for the inhibitory effect on adipogenesis. F-BOX and LRRs interact with several proteins from the PRC1, including S-phase kinase associated protein 1 (SKP1), ring finger protein 1B (RING1B), and BCL6 repressor (BCOR). Similar to the interaction between KDM2B and BCOR, which is necessary to negatively regulate MSC differentiation [97], the knockdown of either *SKP1* or *RING1B* rescues KDM2B-induced adipogenesis inhibition as well as initiates the resumption of the G1/S transition during the second MCE. These results indicate that either SKP1 or RING1B can form a protein complex with KDM2B to mediate adipogenic inhibition. After KMD2B binds to CpG islands, RING1B is recruited to the transcriptional start site of adipocyte genes (e.g., *PPARγ2*) and cell cycle genes, blocking their expression and adipocyte differentiation. This finding suggests that KDM2B forms a noncanonical PRC1 and binds to CpG-rich sequences in intragenic regions of the target loci [96].

Another KDM that appears to control cell cycle progression at the early stage of adipogenic differentiation is KDM4B, which catalyzes the removal of the H3K9me3 and H3K36me3 marks. Gou et al. showed that *KDM4B* is a C/EBPβ target gene required during the early adipogenesis stages of 3T3-L1 cells. This result indicates that C/EBPβ might regulate gene expression through indirect regulation of histone K methylation, which is consistent with the effect of *C/EBPβ* knockdown in impairing KDM4B activity. Furthermore, by coimmunoprecipitation assays, the same authors showed that, similar to C/EBPβ, KDM4B is also recruited to the promoter regions of cell cycle genes, including cell division cycle 45 homolog (*Cdc451c*), mini-chromosome maintenance complex component 3 (*Mcm3*), GINS complex subunit 1 (*Gins*1), and cell division cycle 25 homolog c (*Cdc25c*) 20 h after, but not before, induction. This finding reveals that C/EBPβ upregulates *KDM4B* expression, which in turn forms a complex with C/EBPβ and targets the promoters of the four cell cycle genes, where KDM4B is required to demethylate H3K9me3, thereby enhancing the transcription of cell cycle-related genes [93]. Together, these findings suggest the importance of C/EBPβ in transactivating the promoters of cell cycle genes; however, the role of KDM4B in mediating epigenetic changes in the H3K9me3-heterochromatin structure seems to be crucial for increasing the transcription of the genes described herein, especially since *KDM4B*-depleted cells significantly decrease MCE and suppress the terminal differentiation of 3T3-L1 cells into adipocytes [93].

In addition to its role in cell cycle regulation, it was recently found that KDM4B also stimulates the terminal differentiation of adipocytes. Jang et al. showed that both the mRNA level increase and the KDM4B protein accumulation were correlated with the corresponding levels of C/EBPα and PPARγ during adipogenesis of 3T3-L1 cells. KDM4B stimulates *C/EBP*α and *PPARγ* expression by removing H3K9me2/3 to stimulate adipocyte differentiation [98]. Although these findings have revealed that KDM4B broadly participates in the early and terminal differentiation of the adipogenic phenotype, the role of KDM4B is still debated. For instance, in human MSCs, KDM4B appears to have an opposing action by promoting osteogenesis instead of adipogenic differentiation. In contrast, downregulation of *KDM4B* led to enhanced adipogenic differentiation by increasing *PPARγ* and *CD36* expression [99], while the expression level of *KDM4B* was low in the MSCs from aged mice compared with those from young mice, leading to bone loss in the aging mice by changing the cell fate of the BMSCs toward adipogenesis [99]. More recently, two independent studies showed that the loss of *KDM4B* in adipocytes leads to increased obesity in mice on a high-fat diet [79,100]. In both cases, the cell diameter of the adipocytes was found to increase in the *KDM4B*-knockout mice. Consistent with the fat accumulation, *PPARγ* and *FABP4* were significantly increased in the *KDM4B*-knockout mice. Subsequent analysis also showed that *KDM4B*-deficient mice suffered glucose intolerance and insulin resistance [79,100]. Additionally, the loss of KDM4B activity leads to increased H3K9me3 levels and decreased H4ac levels in genes critical for energy expenditure, including *PPARα* and PPARG coactivator 1*α* (*PGC1α*)*,* suggesting that KDM4B is critical for maintaining whole-body energy homeostasis [100]. Together, these results suggest that, if we are able to modulate KDM4B activity, then we might be able to prevent obesity.

More recently, Brier et al. showed that KDM5 family members (KDM5A-C) also play an essential role in regulating cell cycle genes and the differentiation of preadipocytes [95]. Although KDM5 modulates the levels of H3K4me3 at promoters in 3T3-L1 preadipocytes, its deficiency caused by knockdown does not have a significant effect on gene expression per se. Notably, knocking down *KDM5A/B/C* does not affect the first wave of TF activation (e.g., *C/EBP*β) induced during early adipogenesis but is required for the second wave of TF expression for progression of adipogenic differentiation (*C/EBP*α and *PPARγ*). Interestingly, a genome-wide analysis revealed that KDM5A has a dual role as a coactivator and repressor of transcription [95]. In this regard, the authors showed that the high occupancy of KDM5 promoters and the high levels of H3K4me3 were associated with highly expressed genes, whereas KDM5-repressed promoters had low KDM5-occupancy and low levels of H3K4me3 and promoter activity. Though the molecular basis of the dual role of KDM5 in the regulation of promoter activity is unclear, evidence suggests that it might be controlled in a promoter-dependent manner. For instance, KDM5-activated promoters are not extensively associated with the TATA box but are highly associated with demethylated CpG islands. Further analyses also showed that among the KDM5-activated genes there is a large set of cell cycle regulators and that KDM5 is essential for MCE, as demonstrated by the knockdown of *KDM5* leading to impaired early adipogenesis by interfering with MCE [95].

On the other hand, it was found that KDM4C can negatively affect *PPARγ* expression independent of its demethylase activity [101]. Although *KDM4C* is continually expressed during adipogenic induction, it has been shown by luciferase assays that overexpression of the Tudor domain (a conserved protein structural motif that interacts with other proteins), but not the enzymatic JmjC domain of KDM4C, decreases the adipogenic response of 3T3-cells by decreasing *PPARγ* expression. It appears that transcriptional repression might be mediated by KDM4C binding to histone deacetylases (HDAC1 and HDAC3) as a mechanism to form a transcriptional repression complex. Supporting these findings, the addition of trichostatin A, an HDAC inhibitor, or knockdown of *KDM4C* restored PPARγ activity and the fat storage capacity of the adipocytes [101]. This finding suggests that different domains of KDM4C could have indirect repressive functions by recruiting inhibitory complexes to chromatin, as well as a reader function that differentiates their target in the genome without the requirement for demethylase activity [101,102].

More recently, some studies have shown that KDM6A, also known as UTX, an H3K27me2/3 demethylase, acquires distinct roles during adipogenic differentiation. For instance, KDM6A deficiency in human MSCs results in enhanced adipogenesis by promoting the expression of *PPARγ* and C/EBPα [103]. This finding indicates a negative role for KDM6A in adipogenesis; however, when the KMD6A function in mouse ESCs was damaged, the potential of *KMD6A*-expressing cells to differentiate into adipocytes was notably decreased [104]. Thus, KMD6A can regulate adipogenesis in a stage-dependent manner. Supporting this finding, *KDM6A*-deficient 3T3-L1 (preadipocytes) had enhanced adipogenic differentiation, in contrast to the mESCs that lacked KDM6A [104]. In addition to the upregulation of pluripotent genes (*Nanog*, *Sox2*, and *Pouf1*), the increased expression of *c-Myc*, due to the lack of KDM6A activity in mESCs, prevents adipocyte generation; however, the phenotype can be rescued by adding a Myc inhibitor (10058-f4). In contrast, in preadipocytes, depletion of *KDM6A* in the presence of the same inhibitor resulted in inhibited adipogenesis. These findings suggest that KDM6A enhances adipogenesis in mESCs by inhibiting c-Myc and suppressing adipogenesis in preadipocytes by activating c-Myc, supporting the idea that KDM6A may modulate *c-M*yc expression during adipocyte differentiation in a stage-dependent manner [104].

Another demethylase that catalyzes the demethylation of H3K27me3 is KDM6B. Similar to KDM4B, KDM6B plays an essential role in osteogenic commitment. However, in MSCs, depletion of *KDM6B* promotes adipogenesis in vitro as well as reduction in bone formation in vivo after subcutaneous transplantation onto a bone scaffold [99]. Interestingly, it has been found that KDM6B facilitates the browning of inguinal WAT (iWAT) by resolving the bivalent domains of H3K27me3 and H3K4me3 to activate BAT genes. For instance, compared with mature BAT adipocytes, mature iWAT adipocytes retain a large portion of H3K27me3 in BAT genes. However, the treatment of primary iWAT adipocytes with forskolin or the induction of *KDM6B* overexpression led to a strong reduction in H3K27me3 at BAT genes such as *Ucp1*. These data suggest that BAT-selective genes in iWAT are epigenetically poised for activation, a finding that is in step with that indicating that KDM6B mediates the transition of white fat with high plasticity into brown adipocytes [105].

## 6. Conclusions and Perspectives

**Figure 3:**
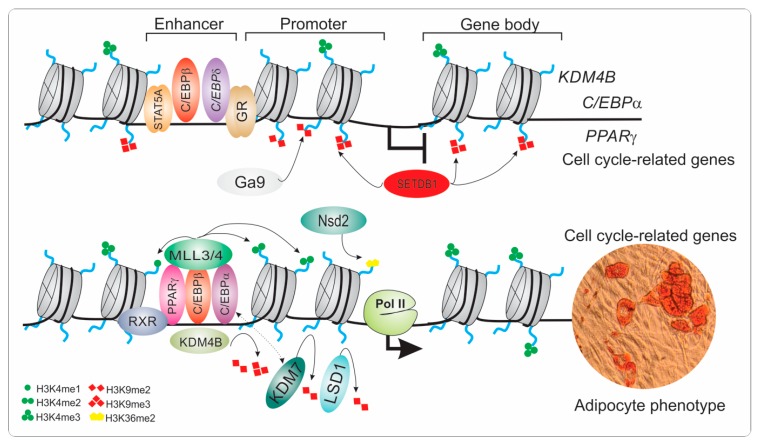
Epigenetic regulation of mesenchymal stem cells during adipocyte differentiation. The differentiation of mesenchymal stem cells into adipocytes is driven by cooperative transcription factors and epigenetic remodelers that act to modulate the chromatin landscape. The recruitment of early adipogenic factors such as C/EBP*β*, C/EBP*δ* as well other transcription regulators (STAT5A, GR) to regulatory elements (enhancers) is mediated by resolving bivalent H3K4me3 and H3K9me3 marks to a monovalent active mark, H3K4me3. The early transcription factor C/EBP*β* stimulates the expression of *KDM4B*, which in turn serves as a cofactor of C/EBP*β* to facilitate the transcriptional activation of cell cycle-related genes by removing H3K9me3 marks and recruiting MLL3/4 to establish active enhancers. In addition, KDM4B and LSD1 remove H3K9me2 and H3K9me3 (deposited by SETDB1 and G9a) on *C/EBPα* and *PPARγ* loci to promote their expression, whereas Nsd2 increases H3K36me2 to activate expression of *C/EBPα* and others adipogenic targets of PPAR*γ*. In addition, the interaction of KDM7 with C/EBP*α* leads to the removal of H3K9me2 from the promoter of *PPARγ* to modulate its expression and that of other adipogenesis-related genes. Together, these mechanisms contribute to shaping the chromatin landscape to induce adipocyte-specific gene expression, thus promoting the adipogenic phenotype. C/EBP: CCAT/enhancer-binding protein; GR: glucocorticoid receptor; H3K4me1: histone H3 lysine 4 monomethylation H3K4me2: histone H3 lysine 4 dimethylation; H3K4me3: histone H3 lysine 4 trimethylation; H3K9me2: histone H3 lysine 9 dimethylation; H3K9me3: histone H3 lysine 9 trimethylation; H3K36me2: histone H3 lysine 36 dimethylation; KDM: histone lysine methyltransferase; LSD1: histone lysine demethylase 1; Nsd2: nuclear receptor binding SET domain protein 2; MLL3/4: mixed-lineage leukemia 3-4; Pol II, RNA polymerase II; PPARγ: peroxisome proliferator-activated-γ; RXR: retinoid X receptor; SETDB1: SET domain bifurcated 1; STAT5A: signal transducer and activator of transcription 5A.

Adipose tissue plays an essential role in energy metabolism and glucose homeostasis. However, increased fat deposition during obesity is a major health concern that has reached alarming proportions worldwide. Recent advances in genome reorganization highlight the role of lysine demethylases to shape permissive chromatin states during adipogenic commitment and adipocyte differentiation (Figure 3).

In addition, emerging evidence shows that misregulation of lysine demethylases, including LSD1, KDM3A, and KDM4B, leads to the development of obesity, glucose intolerance, and insulin resistance (Table 1), suggesting that KDMs play an essential role in maintaining the homeostatic endocrine and metabolic physiology of adipose tissue. In contrast, it has been found that the upregulation of *LSD1* or *KDM6B* can modulate the transition of white fat into brown adipocytes, which can burn excess calories when stimulated. Although these findings provide new insights and could be considered when developing therapeutic targets for preventing or combating obesity, the truth is that adipocyte development is quite complex since they can reside in various tissues such that their modulation may be differentially regulated. Adding to this complexity, adipocyte development can also be modulated in a development stage-dependent manner, which means that precursor cells giving rise to adipocytes during embryogenesis and in adulthood may be established differentially.

The recent finding that a new form of adipocyte can emerge from muscle-cell precursors [80] reinforces the idea that adipocytes have multiple origins. Thus, it is crucial to characterize whether chromatin configuration mediated by lysine demethylases is a generalized process during adipogenic commitment or differs by each adipocyte depot. Moreover, histone demethylases can modify methylation status at specific genomic elements (e.g., bivalent promoters, enhancers, and others) and thus determine cell fate decisions such that it is important to determine whether KDMs are recruited in a spatiotemporal manner, how they orchestrate the activation of cell lineages during adipogenic commitment, and how they repress genes of alternative lineages. In addition, given that histone demethylases have dual substrates, the signals or factors dictating substrate specificity during the establishment and maintenance of adipocyte identity are widely unknown and require further study. Additionally, emerged findings indicate that KDMs may have other functions that are distinct from their demethylase activity, such as those demonstrated by KDM2B and KDM4C acting as molecular scaffolds that enable the recruitment of modifiers that shape chromatin structure (e.g., PRC1 and HDACs). With this in mind, it would be interesting to determine whether other KDMs may suspend their enzymatic activity during adipocyte differentiation. We believe that increasing our understanding of histone lysine demethylases in the regulation of cell fate decisions, a point at which they appear to have a central role, will help us selectively repress local adipogenesis and prevent obesity-related diseases in the future. For these reasons, it is essential to establish the physiological effects of epigenetic modulation during adipogenesis as this knowledge has implications on pathophysiology and/or cellular therapy.

## Authors Contribution

Conceptualization, G.I.N.-C.; Writing-Original preparation, B.A.R.-J., L.M.C.-C., R.P-C., F.J.A.-A.; Writing-Review & Editing, G.I.N.-C., A.Z.-P. and R.A.R.-H. Supervision and design of the images G.I.N.-C.

## Figures and Tables

**Table 1:** Histone lysine demethylases involved in adipogenic development and its relation to obesity.

Chromatin Modifier	Histone Targets	Type of Adipocytes	Roles in Adipose Tissue	Reference
LSD1	H3K4me2, H3K9me2	Brown, Beige, White	The adipose-specific deletion of *LSD1* induces an increase in WAT-selective gene expression, impaired oxidative phosphorylation, reduced energy expenditure and increased fat mass.	[23,84]
KDM4B	H3K9me3	Brown, White	Loss of *KDM4B* impairs energy expenditure, adaptive thermogenesis, and adipose tissue lipolysis, resulting in obesity and associated metabolic dysfunction.	[79,100]
KDM3A	H3K9me1, H3K9me2	Brown, Beige	KDM3A regulates beige and brown adipogenesis by modulating the expression of metabolic genes. The loss of KDM3A function results in obesity and hyperlipidemia in mice.	[78,106]
